# Comparative Transcriptome Analysis Provides Molecular Insights into the Interaction of *Beet necrotic yellow vein virus* and *Beet soil-borne mosaic virus* with Their Host Sugar Beet

**DOI:** 10.3390/v12010076

**Published:** 2020-01-08

**Authors:** Jose Fernando Gil, Daniel Wibberg, Omid Eini, Eugene I. Savenkov, Mark Varrelmann, Sebastian Liebe

**Affiliations:** 1 Department of Plant Biology, Uppsala BioCenter SLU, Swedish University of Agricultural Sciences, Linnean Center for Plant Biology, 75007 Uppsala, Sweden; jose.fernando.gil@slu.se (J.F.G.); eugene.savenkov@slu.se (E.I.S.); 2 Genome Research of Industrial Microorganisms, CeBiTec, Bielefeld University, D-33501 Bielefeld, Germany; dwibberg@cebitec.uni-bielefeld.de; 3 Department of Plant Protection, School of Agriculture, University of Zanjan, Zanjan 45371-38791, Iran; omid.eini@znu.ac.ir or; 4 Department of Phytopathology, Institute of Sugar Beet Research, 37079 Göttingen, Germany; varrelmann@ifz-goettingen.de

**Keywords:** auxin, *Benyvirus*, plant hormone, pathogenesis related protein, resistance, salicylic acid, transcriptome

## Abstract

*Beet necrotic yellow vein virus* (BNYVV) and *Beet soil-borne mosaic virus* (BSBMV) are closely related species, but disease development induced in their host sugar beet displays striking differences. *Beet necrotic yellow vein virus* induces excessive lateral root (LR) formation, whereas BSBMV-infected roots appear asymptomatic. A comparative transcriptome analysis was performed to elucidate transcriptomic changes associated with disease development. Many differentially expressed genes (DEGs) were specific either to BNYVV or BSBMV, although both viruses shared a high number of DEGs. Auxin biosynthesis pathways displayed a stronger activation by BNYVV compared to BSBMV-infected plants. Several genes regulated by auxin signalling and required for LR formation were exclusively altered by BNYVV. Both viruses reprogrammed the transcriptional network, but a large number of transcription factors involved in plant defence were upregulated in BNYVV-infected plants. A strong activation of pathogenesis-related proteins by both viruses suggests a salicylic acid or jasmonic acid mediated-defence response, but the data also indicate that both viruses counteract the SA-mediated defence. The ethylene signal transduction pathway was strongly downregulated which probably increases the susceptibility of sugar beet to *Benyvirus* infection. Our study provides a deeper insight into the interaction of BNYVV and BSBMV with the economically important crop sugar beet.

## 1. Introduction

*Beet necrotic yellow vein virus* (BNYVV) and *Beet soil-borne mosaic virus* (BSBMV) are closely related members of the genus *Benyvirus* within the family Benyviridae [1]. *Beet necrotic yellow vein virus* is the causal agent of rhizomania disease in sugar beet and is distributed worldwide [2], whereas BSBMV occurs only in sugar beet growing areas in the United States [3,4]. Both viruses are transmitted by the soil-borne plasmodiophoromycete *Polymyxa betae* that infects the root tissue of young sugar beet plants [5,6]. Resting spores of the plasmodiophoromycete containing infectious virus particles can survive in the soil for decades. Although, both viruses are closely related species with a similar host range and the same vector species, symptoms induced in their major host plant sugar beet show marked differences [7]. Roots of BNYVV-infected plants display severe disease symptoms including reduced size, deformation, and necrosis of vascular tissue. Extensive proliferation of lateral roots (LRs) leading to a root beard is the characteristic feature of rhizomania disease. The symptoms in systemically infected leaves are characterized by vein yellowing and necrosis but are seldom observed in the field. In contrast, symptoms of BSBMV-infected plants are mainly restricted to leaves that display light yellow vein banding, mottling, mosaic patterns, and growth disorders. Infected roots appear asymptomatic in the field. Therefore, yield losses caused by BSBMV are very low [8] and probably of minor economic importance. 

Both BNYVV and BSBMV are multipartite, single-stranded, positive-sense RNA viruses which have four capped polyadenylated RNA components separately encapsidated in rod-shaped particles. Both viruses display similar genome organisation, but sequence differences allow their classification into two different species [1,4]. However, a high level of sequence conservation and sequence similarity between BNYVV and BSBMV suggest functional similarity among corresponding genes of both viruses. Moreover, the genomic components can be exchanged between both viruses, resulting in viable reassortants [4,9,10]. The RNA1 contains one open reading frame (ORF) encoding a replicase protein that harbours motifs for methyltransferase, helicase, papain-like protease and RNA-dependent RNA polymerase. The first ORF on RNA2 encodes the coat protein (CP) and the minor CP read-through protein (CP-RT) involved in vector transmission [11]. The next three overlapping ORFs of RNA2 form the triple gene block (TGB1-3) responsible for cell-to-cell movement of the virus [12]. The last ORF of RNA2 encodes a cysteine-rich protein (P14), a viral suppressor of RNA silencing [13,14]. The BNYVV RNA3 is required for long distance movement in *Beta* species [15] and encodes the P25 virulence factor of the virus responsible for the rhizomania disease development in sugar beet [16]. Similarly, BSBMV RNA3 is also involved in long distance movement of the virus but encodes a 29 kDa protein (P29) that has only a 23% amino acid sequence similarity to the P25 protein [10]. The RNA4-encoded BNYVV P31 and BSBMV P32 proteins, respectively, are necessary for efficient vector transmission of the viruses [17,18].

Despite their close relationship, both viruses display striking differences in disease development indicating a virus specific interaction with their host sugar beet. Extensive proliferation of LRs induced upon BNYVV infection requires the presence of the P25 virulence factor [16]. The LR formation is a developmental process tightly controlled by the phytohormone auxin (indole-3-acetic acid, IAA) and its transport and signalling components [19]. Recently, we have shown that BNYVV hijacks auxin-regulated pathways in sugar beet by the interaction of P25 with the auxin/indole acetic acid protein (AUX/IAA) BvAUX28 [9]. At low auxin concentrations, AUX/IAAs act as transcriptional repressors by suppressing the activity of auxin response factors (ARFs), transcriptional activators of auxin-responsive genes [20]. Elevated auxin concentrations promote the degradation of AUX/IAAs by the SCF^TIR1^ multiprotein complex [21,22,23], leading to the derepression of ARFs which, in turn, regulate the expression of genes involved in LR development. Several AUX/IAA–ARF transcriptional modules, which regulate LR development sequentially, have been identified [24]. BNYVV P25 inhibits the transcriptional repressor activity of BvAUX28, probably, by interfering with its nuclear localization [9]. The analysis of gene expression showed that several genes encoding lateral organ boundaries domain (LBD) transcription factors (TFs) and expansins (EXP) are highly upregulated during BNYVV infection [9]. Expression of *LBDs* is regulated by AUX/IAA–ARF modules and play a central role in LR development by the activation of *EXPs* which encode cell wall loosening proteins involved in many aspects of plant development including LR formation [25]. Notably, the expression pattern of certain *LBDs* and *EXPs* was altered only in the presence of BNYVV but remained unchanged in response to BSBMV infection [9]. This suggests that BSBMV does not interact with auxin-regulated pathways to the extent leading to the root disease symptoms typical for BNYVV infections. 

Previous studies in *Arabidopsis thaliana* and *B*. *vulgaris* identified several differentially expressed genes (DEGs) associated with LR formation and stress signalling in response to BNVVV suggesting a strong reprogramming of the host transcriptome during rhizomania disease development [26,27]. However, to the best of our knowledge, there are no studies applying next-generation sequencing (NGS) technologies for global transcriptome profiling of BNYVV- and/or BSBMV-infected sugar beet plants. The current study contributes to filling in this gap. Benyviruses are known to occur mostly in mixed infections with the two *Pomovirus*, *Beet soil-borne virus* and *Beet virus Q*, that are also transmitted by *P*. *betae* [28]. This would make transcriptomic analysis of naturally infected sugar beet plants (soil-mediated infection) inconclusive, because the effect of other viruses and the vector on the host transcriptome cannot be excluded. To circumvent this problem, we used full-length infectious cDNA clones [29] to establish single infections of BNYVV and BSBMV in sugar beet. This approach yielded transcriptomic data characterizing solely the response of sugar beet to infections with BNYVV and BSBMV in a compatible interaction using a susceptible genotype. These transcriptomic data were analysed with particular focus on alterations of expression of the genes involved in auxin signalling, LR development and plant defence to characterize disease development in BNYVV- and BSBMV-infected plants at the transcriptional level. The study provides, for the first-time, insights into the interaction of BNYVV and BSBMV with the economically important crop sugar beet and highlights commonalities and differences in response to these closely related Benyviruses.

## 2. Materials and Methods

### 2.1. Plant Material and Virus Inoculation

Infectious full-length clones of BNYVV and BSBMV [29] were used for inoculation of sugar beets. A susceptible (KWS03) sugar beet genotype was provided by KWS Saat SE (Einbeck, Germany). Young sugar beet seedlings were infected using a previously developed protocol [30] with slight modifications. First, plants of *Beta macrocarpa* were agroinfected with both virus infectious clones when the first two leaves (BBCH12) were fully developed. One true leaf and both cotyledons were inoculated with *Rhizobium radiobacter* (syn. *Agrobacterium tumefaciens*) strain GV2260 with an OD_600_ = 0.5 [31]. Plants were kept for symptom development in a climate chamber at day and night temperatures of 24 °C and 18 °C, respectively, and a 14 h photoperiod. Leaves displaying systemic symptoms were harvested (1 g) and grinded in 4 mL 0.05 M phosphate buffer (1 g of KH_2_PO_4_ and 7.2 g of Na_2_HPO_4_ in 1 L of diethyl pyrocarbonate (DEPC)-treated water, pH 7 to 7.4). The plant sap was added to a 15 mL conical centrifuge tube (Sarstedt AG & Co. KG, Nümbrecht, Germany) containing six sugar beet seedlings (7 days old) and 40 mg of carborundum (Sigma–Aldrich Chemie GmbH, Hamburg, Germany). The tube was then mixed on a Vortex Genie 2 (Scientific Industries) at maximum speed for 50 s and then upside down for 10 s. Plants were left for an additional 5 min in the inoculum suspension. Seedlings were washed in tap water and planted in sterile soil and covered for two days with plastic bags. All plants were grown as described above in a climate chamber for 44 days. For transcriptome sequencing, 200 mg of root tissue including LRs was harvested from each plant, immediately frozen in liquid nitrogen and stored at −80 °C until RNA extraction. Additionally, an enzyme-linked immunosorbent assay (ELISA) was applied to measure the virus content in LRs (100–150 mg) of sugar beets. Antibodies specific for BNYVV and BSBMV were obtained from the Leibniz Institute DSMZ-German Collection of Microorganisms and Cell Cultures (Braunschweig, Germany). The assay was conducted according to the manufacturer’s instructions. Virus quantification was conducted with a Bradford assay as described previously [32].

### 2.2. RNA Extraction, Library Preparation and Sequencing

Two hundred milligrams of root tissue was grinded in 1 mL TRIzol Reagent (Invitrogen, Carlsbad, CA, USA) using mortar and pestle. Grinded samples were incubated at room temperature for 5 min and centrifuged at 12,100× *g* for 10 min (4 °C) to remove crude plant material. Approximately 700 µL of the supernatant was mixed with an equal volume of absolute ethanol (−20 °C). The total RNA was then purified using the Direct-zol RNA Miniprep Plus (Zymo Research, Orange, CA, USA) following the manufacturer’s instructions. Dual cDNA libraries were prepared, sequenced and analysed as recently described [33] with smaller modifications. In brief, 2.25 μg of RNA per sample was used for preparation of stranded libraries with the TruSeq mRNA Sample Preparation Kit (Illumina Inc., San Diego, CA, USA). Sequencing of the prepared cDNA libraries was carried out on the Illumina HiSeq 1500 platform (Illumina Inc., San Diego, CA, USA). To enable sufficient transcriptome coverage as described before [34], the 15 cDNA libraries were sequenced in a single read and rapid mode run with 71 cycles to end up in an approximately coverage of 100× per beet gene. Data analysis and base calling was accomplished by an in-house pipeline based on CASAVA 1.8.2. [35]. The raw data for all sequencing libraries were deposited on the EBI ArrayExpress server, accession E-MTAB-8187.

### 2.3. Transcriptome Analysis

The sequenced reads were trimmed by applying Trimmomatic 0.36 [36]. Data for each replicate were subsequently mapped to the virus genomes of BSBMV (KX352033, KX352170, KX352171, KX352034) and BNYVV (KX665536, KX665537, KX665538, MF476800) using Bowtie 2 [37]. Two mismatches were allowed to account for possible sequencing errors. Unmapped reads of this approach were then used for another mapping against the sugar beet genome RefBeet 1.2.2. by applying TopHat2 [38]. The resulting filtered BAM files (option –f 4) were imported in the RNAseq analysis platform ReadXplorer 2.2.3. [39,40] for the visualization and analysis of short read alignments. The R package DESeq2 [41] was selected as the method for the differential analysis. In our study, genes with a minimum log2 fold change of 2, and a *p*-adjusted value smaller than 0.05 were classified as significantly expressed genes (DEGs). In addition, reads per kilobase per million reads (RPKM) values were calculated from exported read count tables, using the single best match option for each of the separate libraries. Probability calculations were omitted for the RPKM calculation, as these values were not used to determine differential significant differences among the datasets but merely depicted general levels of transcription. Gene set enrichment analysis was performed using KOBAS 3.0 to identify significantly enriched gene ontology (GO) categories and KEGG (Kyoto Encyclopedia of Genes and Genomes) [42,43,44]. Identification of transcription factors and regulatory networks within the DEGs was conducted using the database PlantTFDB 4.0 [45,46,47]. Sequence alignments and phylogenetic analysis were done with MEGA7 using default options [48].

### 2.4. RT-qPCR

The expression of twelve genes was measured by RT-qPCR to validate the transcriptomic data. The genes were manually selected in order to cover the range of up-, down- and non-regulated genes. Primers for target genes (Supplementary Materials Table S1) were designed using Primer-BLAST [49]. Sugar beet *glycerinaldehyd-3-phosphat-dehydrogenase* and *elongation factor 1-delta* were used as reference genes. Total RNA was extracted from three independent biological replicates as described above, and 2 µg of total RNA was reverse transcribed into cDNA using RevertAid Reverse Transcriptase (Thermofisher, Waltham, MA, USA). The cDNA was diluted 1:5 in water prior to its application in RT-qPCR. The reaction was set up in a 15 µL volume containing 1× iTaq Universal SYBR Supermix (BioRad), 0.330 µM of each primer and 2 µL cDNA. The RT-qPCR was carried out in the CFX96 Real Time System C1000 Touch Thermal Cycler (Bio-Rad, Feldkirchen, Germany). The reaction was set with initial denaturation of 95 °C for 3 min followed by 40 cycles of 95 °C for 30 s, 60 °C for 20 s, 72 °C for 30 s, final extension of 72 °C for 5 min. Each biological sample was analysed in two technical repeats. Data normalization and calculation of relative expression values were done using the 2^−ΔΔCt^ method [50].

## 3. Results

### 3.1. Sequencing Statistic and RT-qPCR Validation

**Figure 1:**
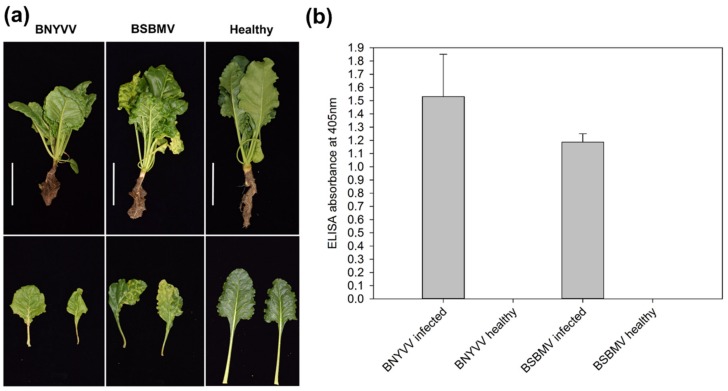
Symptoms and virus content of plants used for transcriptome analysis. (**a**) Typical symptoms of *Beet necrotic yellow vein virus* (BNYVV) and *Beet soil-borne mosaic virus* (BSBMV) infected plants from a susceptible genotype compared to healthy plants. The white bar represents the scale (10 cm). (**b**) Absorbance values (405 nm) measured in an enzyme linked immunosorbent assay (ELISA) on lateral roots of BNYVV- and BSBMV-infected plants (*n* = 3) compared to healthy plants. The error bars represent the standard deviation.

Plants subjected to RNA-seq were selected based on symptom development and virus content (Figure 1). In total, about 339 million sequence reads, amounting to 24 Gb of sequence information, were generated for all transcriptome libraries (Table 1). Between 4 and 7% of all reads obtained from plants infected with BNYVV were mapped to the virus genome, whereas approximately 83% of all reads originated from sugar beet. For BSBMV, the amount of virus reads was higher (23–25%) and the number of reads mapped to sugar beet genome ranged between 62 and 63%. Approximately 15% of the reads in each sample could be neither mapped to the sugar beet nor to the virus genomes. Reads of other known viruses were not found, but several unknown transcripts with unusual splicing events were identified among the unmapped reads. To validate the sequencing data, the expression of 12 sugar beet genes was measured by RT-qPCR. The genes were manually selected in order to cover the range of up- and downregulated genes as well as genes with no significant change in the expression (Supplementary Materials Table S2). The log2 fold change did not always match the transcriptome data, but the expression pattern was very similar indicated by a high Pearson correlation coefficient (*r* = 0.95).

### 3.2. Functional Classification of DEGs

**Figure 2:**
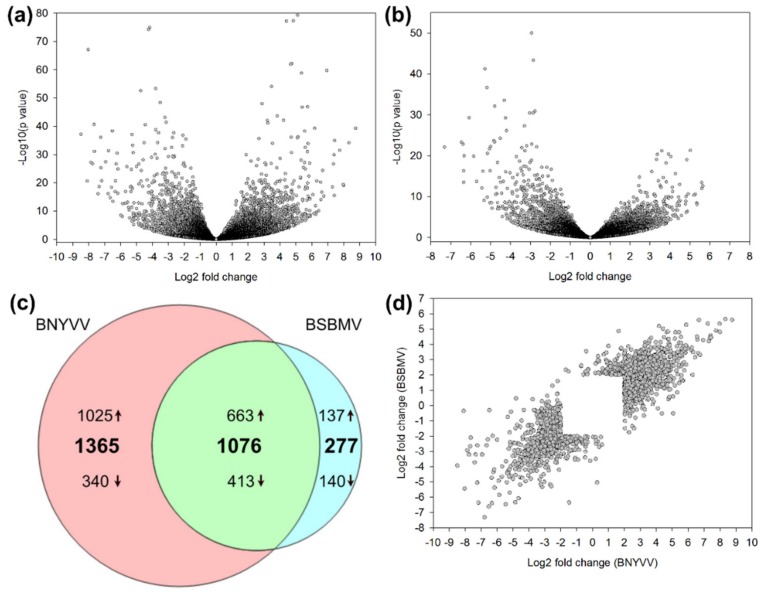
Analysis of differentially expressed genes (DEGs) in BNYVV- and BSBMV-infected plants from a susceptible genotype. Volcano plots display log2 fold change plotted against the *p*-values (−log10) of all genes expressed in BNYVV (**a**) and BSBMV (**b**) infected plants. The Venn diagram shows the comparison of up- and downregulated DEGs between BNYVV and BSBMV (**c**). The log2 fold change of DEGs from BNYVV and BSBMV were plotted against each other (**d**).

In total, the expression of 22,595 and 22,335 genes could be measured in BNYVV (Figure 2a) and BSBMV (Figure 2b) infected plants, respectively. After filtering (*p*-value < 0.05), 8004 genes were differentially expressed in the BNYVV dataset (*p*-value < 0.05) (Supplementary Materials Table S3) and 5037 genes in the BSBMV dataset (Supplementary Materials Table S4). In order to focus on the most important genes, we further narrowed down the number of DEGs by applying a stringent threshold for the log2 fold change (>2 or <−2). This resulted in 2441 DEGs within the BNYVV dataset (1688 upregulated; 753 downregulated) and 1353 DEGs in the BSBMV dataset (800 upregulated; 553 downregulated). Among the DEGs identified in BNYVV (Supplementary Materials Table S5) and BSBMV (Supplementary Materials Table S6) infected plants, only 1076 genes (663 upregulated; 413 downregulated) were differentially expressed in both datasets (Figure 2c). A total of 1365 DEGs (1025 upregulated; 340 downregulated) were exclusively found in the BNYVV dataset, whereas the BSBMV dataset contained only 277 specific DEGs (137 upregulated; 140 downregulated). We then merged both datasets to obtain one dataset containing all DEGs from BNYVV and BSBMV. The expression values from BNYVV and BSBMV were plotted against each other to get an overall view of the correlation among both datasets. The expression pattern followed a similar trend as indicated by the linear shape of the point cloud (Figure 2d).

The GO classification resulted in 20 significantly enriched GO terms within the BNYVV dataset (Table 2). The same GO terms also ranked highest in the BSBMV dataset, but only 12 of them were significantly enriched. Although BNYVV and BSBMV displayed a similar trend in GO classification, the total number of DEGs mapped to the different GO terms was always higher for BNYVV. As expected, both datasets contained a high number of up- and downregulated DEGs classified in the GO terms “response to stimulus” and “response to stress” indicative for plant defence. Interestingly, 128 DEGs from BNYVV-infected plants (112 upregulated; 16 downregulated) were associated with “cell wall organization or biogenesis”, whereas only 57 DEGs (50 upregulated; 7 downregulated) from BSBMV-infected plants mapped to this GO term (Table 2). The DEGs were also classified using the Kyoto Encyclopedia of Genes and Genomes (KEGG) database to identify enriched pathways (Table 3). The DEGs from both viruses mapped to the same KEGG pathways, but 14 were significantly enriched in BNYVV-infected plants and only three in BSBMV-infected plants. Similar to the GO analysis, the total number of DEGs mapped to the different pathways was always higher for BNYVV. The pathways “phenylpropanoid biosynthesis”, “biosynthesis of secondary metabolites” and “metabolic pathways” were significantly enriched in both BNYVV- and BSBMV-infected plants, although more DEGs from BNYVV were mapped to these pathways. Within the phenylpropanoid biosynthesis pathway, the expression of many enzymes catalysing the production of secondary metabolites (i.e., caffeoyl-CoA, ferulic acid, scopolin) was also upregulated by BNYVV (Supplementary Materials Figure S1) and BSBMV (Supplementary Materials Figure S2). Interestingly, both viruses upregulated the expression of various peroxidases involved in the production of different lignin compounds including p-hydroxyphenyl lignin, guaiacyl lignin, 5-hydroxy-guaiacyl lignin and syringyl lignin.

### 3.3. Interaction with Auxin Biosynthesis and Signalling Pathways

**Figure 3:**
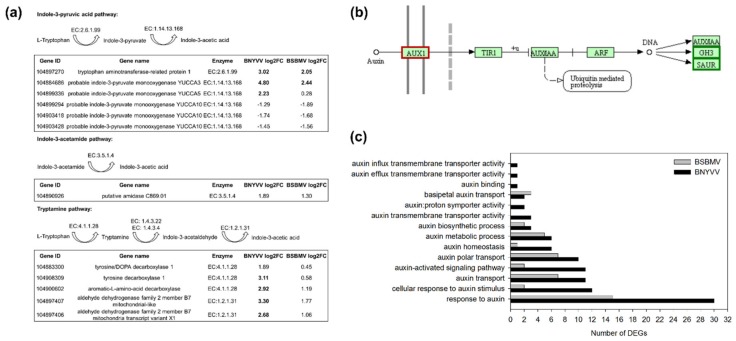
Interaction of BNYVV and BSBMV with auxin biosynthesis and auxin signalling pathways in sugar beet (susceptible genotype). (**a**) Log2 fold change (log2FC) of genes encoding proteins involved in the biosynthesis of auxin in sugar beet. Enzymatic steps of the indole-3-pyruvic acid, indole-3-acetamide and tryptamine pathways in sugar beet were retrieved from the Kyoto Encyclopedia of Genes and Genomes database (KEGG). DEGs above the threshold (log2 fold change > 2 or < −2, *p*-value < 0.05) are highlighted in bold letters. (**b**) Auxin signal transduction pathway of sugar beet retrieved from the KEGG database. Components of the pathway are highlighted in red when only DEGs from BNYVV were mapped, whereas green indicates that DEGs from both viruses mapped to this component. (**c**) Number of DEGs belonging to gene ontology (GO) categories related to auxin signalling pathways.

One of our major objectives was to elucidate changes in the host transcriptome that are responsible for the development of rhizomania root symptoms. The massive proliferation of lateral roots requires an activation of auxin biosynthesis pathways resulting in an increase in the cellular auxin level. The l-tryptophan-dependent pathways are supposed to be the main routes for auxin biosynthesis including at least three different pathways, namely, (I) indole-3-pyruvic acid (IPyA), (II) indole-3-acetamide (IAM) and (III) tryptamine (TRA) pathway [51]. We analysed the expression of genes encoding catalytic enzymes belonging to these pathways using the KEGG database (Figure 3a).

Within the IPyA pathway, *tryptophan aminotransferase-related protein 1* was upregulated in BNYVV (3.02) and BSBMV (2.05) infected plants. It encodes an enzyme which converts l-tryptophan to indole-3-pyruvate. Several *YUCCA* genes responsible for the last enzymatic step from indole-3-pyruvate to indole-3-acetic acid were also differentially expressed. Especially, *YUCCA3* was highly upregulated in BNYVV-infected plants (4.80) and also induced by BSBMV (2.44). In contrast, three gene loci of *YUCCA10* were slightly downregulated in both datasets. The IAM pathway remained unchanged by both viruses. The strongest activation of gene expression was observed along the TRA pathway. The expression of *tyrosine decarboxylase 1* necessary for the conversion of l-tryptophan to tryptamine was strongly induced by BNYVV (3.11) but not affected by BSBMV (0.58). Other genes required for the subsequent enzymatic steps within this pathway were also more upregulated in BNYVV-infected plants compared to BSBMV (Figure 3a). The strong activation of the auxin biosynthesis pathways in BNYVV-infected plants must require a higher demand for the precursor l-tryptophan. Indeed, *tryptophan synthase beta chain 2* was more strongly induced in BNYVV (4.79) infected plants compared to BSBMV (2.30). The encoded protein is a subunit of the tryptophan synthetase that catalyses the final two steps in the biosynthesis of l-tryptophan.

The strong activation of auxin biosynthesis prompted us to analyse the signal transduction pathway responsible for regulation of auxin-responsive genes (Figure 3b). The *auxin transporter-like protein 2* (*LAX2*) involved in proton-driven auxin influx was differently expressed in BNYVV-infected plants (2.12) and below the threshold in BSBMV-infected plants (1.37). Genes belonging to the *SAUR* (small auxin up RNA) and *GH3* (glycoside hydrolase) families are primary auxin response genes, which are transcriptionally regulated by auxin. The *indole-3-acetic acid-amido synthetase GH3-1* (*GH3-1*) belonging to the *GH3* gene family was strongly downregulated by both viruses (BNYVV: −3.04; BSBMV: −5). The protein conjugates amino acids to free IAA [52]. Conjugated IAA is biologically inactive and is crucial for auxin homeostasis by inactivating IAA, serving as a reservoir for IAA. Within the *SAUR* gene family, three DEGs were upregulated and one DEG was strongly downregulated by both viruses. Since not all genes are annotated in the KEGG database, we also analysed the results from GO classification to identify more auxin-responsive genes (Figure 3c). Most genes were categorized within the GO term “response to auxin” with 30 DEGs from BNYVV and 15 DEGs from BSBMV. Several GO terms like “cellular response to auxin stimulus”, “auxin-activated signalling pathway” and “auxin homeostasis” were overrepresented only in the BNYVV dataset. Moreover, several DEGs specific to BNYVV were identified (Supplementary Materials Table S7). The gene *pin-likes 3-like* encoding a PIN protein displayed a strong downregulation only in BNYVV-infected plants (BNYVV: −3.36; BSBMV: −0.73). It is involved in the directional cell-to-cell polar auxin transport as an auxin efflux carrier [53]. Furthermore, *WUSCHEL-related homobox 5* (*WOX5*) was also upregulated by BNYVV (3.17). The protein is involved in the specification and maintenance of stem cells in the root apical meristem. Similarly, the expression of the TF *baby boom* (*BBM*) was induced only in BNYVV-infected plants (2.91). The protein promotes cell proliferation, differentiation and morphogenesis, especially during embryogenesis [54]. The N-acetyltransferase *HLS1* was strongly induced upon infection with BNYVV (5.68) and slightly upregulated in BSBMV-infected plants (1.6). In Arabidopsis, HLS1 negatively regulates the expression of *AUR3* encoding a GH3-like protein [55] which conjugates free IAA with amino acid [52,56]. Thus, the high upregulation of *HSL1* is in accordance with the downregulation of *GH3.1* as described above and probably promotes a higher level of free IAA.

### 3.4. Activation of Auxin-Regulated LR Development

Having confirmed the alteration of auxin biosynthesis and signalling mostly in response to BNYVV, but not to BSBMV, the further analyses were focused on the genes involved in LR development. The initiation of LR development in pericycle cells is governed by the auxin-responsive expression of *LBD16*, *LBD18* and *LBD33* TFs [57]. Within our dataset, 8 LBD TFs were identified that showed a virus specific expression pattern (Table 4). Several LBDs, namely, *LBD6*, *LBD15*, *LBD18*, *LBD19* and *LBD33*, were strongly upregulated during BNYVV infection. None of these LBDs were induced in BSBMV-infected plants. Furthermore, *LBD20* and *LBD40* were repressed by both viruses and *LBD21* only by BSBMV. For LR initiation, pericycle cells need to undergo cell division which requires cell cycle reactivation. The TF E2Fa is an essential component that regulates the cell division of pericycle cells and its expression is activated by LBD18/LBD33 dimers [58]. Thus, the induction of *LBD18* and *LBD33* coincide with the upregulation of the cell cycle regulator *E2Fa* (2.4) in BNYVV-infected plants, whereas BSBMV induced only moderate transcriptional changes (1.6; below threshold) of *E2Fa* expression. The LR development in BNYVV-infected plants is also accompanied by highly dynamic changes in *EXP* expression [9] which are, in part, activated by LBD18 [59,60]. Within our dataset, the expression pattern of *EXPs* in BNYVV- and BSBMV-infected plants was very similar, although transcriptional changes were more pronounced in BNYVV-infected plants with a higher number of genes above the applied threshold (Table 4). The highest upregulation was observed for *EXPB15L* (BNYVV: 6.31; BSBMV: 3.48), whereas *EXLA3b* was strongly downregulated (BNYVV: −6.57; BSBMV: −2.22).

### 3.5. Reprogramming of the Plant Transcriptional Network

The high number of DEGs identified in BNYVV- and BSBMV-infected plants was indicative for a strong response of the host transcriptome to the virus infection. This prompted us to analyse the interaction of BNYVV and BSBMV with the plant transcriptional network. To this end, DEGs encoding TFs were identified and assigned to families using the plant transcription factor database (Table 5).

In total, DEGs belonging to 34 different TF families were identified indicating a strong reprogramming of the transcriptional network. Although both viruses strongly influenced the expression of many TFs (Supplementary Materials Table S5), more up- and downregulated TFs were found in BNYVV-infected plants. We also performed an enrichment analysis to identify TFs which possess significantly over-represented targets in the DEG dataset (Supplementary Materials Table S8). Fourteen and 3 TFs displayed a specific enrichment in BNYVV- and BSBMV-infected plants, respectively. The AP2-like ethylene-responsive TF *BBM* was exclusively upregulated by BNYVV (2.91) with the highest number of potential targets. Similarly, the upregulation of *WUSCHEL* (4.62) was specific to BNYVV only. Among the BSBMV DEGs, no potential targets were identified for NAC domain-containing protein 43, NAC domain-containing protein 76 and MADS-box transcription factors 23, indicating a specific role during BNYVV infection. We then analysed TFs belonging to gene families associated with plant defence [61,62], namely, *AP2*, *bHLH*, *bZIP*, *ERF*, *MYB*, *NAC* and *WRKY*. In both datasets, plant defence-related TFs were highly overrepresented (Supplementary Materials Table S9). Within these families, 27 DEGs were identical between both datasets, 46 DEGs were specific to BNYVV, and only 13 to BSBMV, indicating a virus-specific response of sugar beet. Twenty-five DEGs (16 upregulated; 9 downregulated) and 11 DEGs (5 upregulated; 6 downregulated) could be assigned to the *bHLH* family for BNYVV and BSBMV, respectively. The highest upregulated TF was *PIF3* in BNYVV-infected plants (4.22) but not in BSBMV-infected plants (−0.13). The *ERF* family encoding ethylene responsive TFs comprised 16 DEGs (11 upregulated; 5 downregulated) for BNYVV and 11 DEGs (4 upregulated; 6 downregulated) for BSBMV. The gene *ERF098* was highly upregulated in BNYVV-infected plants (5.57), and *ERF017* displayed the highest upregulation in BSBMV-infected plants (2.99). Furthermore, several TFs within this family were exclusively up- and downregulated by BNYVV. Similarly, many TFs belonging to the gene families *MYB*, *NAC* and *WRKY* were found to be upregulated only in BNYVV-infected plants.

### 3.6. Plant Defence Response Against BNYVV and BSBMV

**Figure 4:**
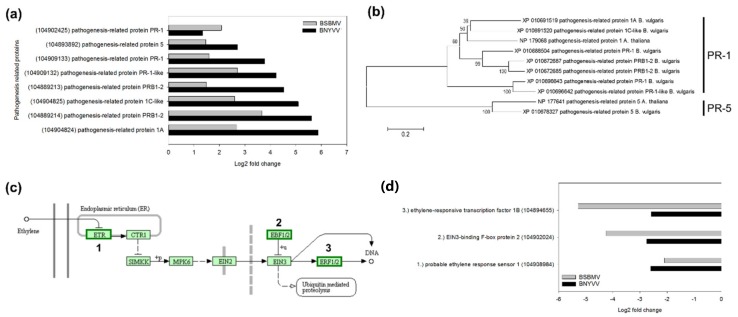
Analysis of DEGs associated with plant defence in sugar beet (susceptible genotype). (**a**) Log2 fold change of upregulated pathogenesis-related proteins (PR) in BNYVV- and BSBMV-infected plants. Brackets indicate the NCBI accession numbers. (**b**) Neighbour-joining phylogenetic tree of upregulated pathogenesis-related proteins during BNYVV and BSBMV infection. Amino acid sequences were retrieved from NCBI and PR-1 and PR-5 from *Arabidopsis thaliana* was included in the tree. (**c**) Ethylene signal transduction pathway of sugar beet retrieved from the KEGG database. Green highlighted components indicate that downregulated DEGs from both viruses were mapped. (**d**) Log2 fold change of DEGs mapped to the ethylene signal transduction pathway.

The large number of differentially expressed TFs related to plant defence was indicative for a strong defence response in sugar beet. In total, 8 highly upregulated PR genes were identified in both datasets (Figure 4a). Except for *PR*-*1*, the expression of PR genes was always higher in BNYVV-infected plants. The gene *PR-1A* displayed the highest expression values in BNYVV-infected plants (5.87), whereas *PRB1-2* was highest in BSBMV-infected plants (3.66). Since activation of the PR protein expression is indicative for a salicylic acid (SA) mediated defence response, we analysed the SA pathway using the KEGG database. The nonexpressor of pathogenesis-related genes 1 (NPR1) and TGA TFs are key regulators of the SA-dependent defence response [63,64,65,66,67]. The expression of the *NPR1* homolog gene in sugar beet was not altered (BNYVV: −0.63; BSBMV −0.48), but *TGA4* was downregulated in both datasets (BNYVV: −2; BSBMV: −2.81). The protein TGA4 probably acts as a negative regulator of the SA and H_2_O_2_ defence response in *A*. *thaliana* [68]. Furthermore, three different gene loci encoding salicylic acid-binding protein 2 (SABP2) were downregulated in BNYVV (ranging between −1.38 and −4.5) and BSBMV (ranging between −0.2 and −3.63) infected plants. SABP2 is required for the conversion of methyl salicylate (MeSA) to SA as part of the signal transduction pathways [69].

Jasmonic acid (JA) can also induce the expression of PR proteins in crosstalk with SA [70,71], but the JA pathway was not altered by both viruses. Apart from SA and JA, ethylene (ET) can also act as a strong signal elicitor in the activation of PR genes [72,73]. The expression of several ethylene responsive TFs was altered during BNYVV and BSBMV infection, indicating an interaction with the ET pathway (Figure 4c). The *ethylene response sensor 1* (*ERS1*) involved in ET perception [74] was repressed to a similar extent by both viruses (Figure 4d). The EIN3-binding F-box protein 2 (EBF2) acts downstream of ERS1 and promotes, in conjunction with EBF1, the degradation of ethylene insensitive 3 (EIN3) [75]. The protein EIN3 plays a central role as a positive regulator of ET perception and transcriptional activator of *ERF* genes [76]. The expression of *EBF2* was more downregulated by BSBMV (−4.25) compared to BNYVV (−2.75), but the expression of *EIN3* remained unaffected by both viruses. The expression of *ethylene-responsive transcription factor 1B* (*ERF1B*) is regulated downstream of EIN3 and was more repressed by BSBMV (−5.28) compared to BNYVV (−2.59). The gene *ERF1B* has a high sequence similarity to *ERF1* from *A*. *thaliana* which is likely the transcriptional activator of the PR gene *PDF1.2* [76,77].

## 4. Discussion

Previous deep sequencing studies on global transcriptomic changes induced by Benyviruses are limited to BNYVV and focused only on the characterization of the host response in leaves of experimental host plants [78,79]. Thus, the results of our study performed in sugar beet roots—the organ where rhizomania disease is manifested—greatly extend the knowledge on Benyviruses. For the identification of DEGs, during data analysis we applied a stringent threshold (log2 fold change > 2 or < −2, *p*-value < 0.05) which has been used in similar transcriptome studies investigating host–pathogen interaction [80,81,82]. It must be emphasized that some DEGs with a *p*-value below 0.5 were designated as non-differentially expressed due to the threshold for the log2 fold change. However, we used this strategy to focus on the most important genes. In general, the expression pattern of DEGs identified in both datasets mostly showed a similar tendency with a large number of DEGs shared by both viruses. The proteins encoded by the housekeeping genes of RNA1 and RNA2 genomic components, which are responsible for replication, encapsidation, cell-to-cell movement and suppression of RNA silencing, share a high amino acid identity among the two viruses [4]. Therefore, it seems likely that the principle mechanisms of virus infection are highly similar as reflected by a set of core genes with altered expression upon infection with both viruses (1076 DEGs).

The dramatic changes in taproot development require a virus specific reprogramming of the taproot transcriptome as, indeed, indicated by the remarkably high number of BNYVV-specific DEGs found in this study. The extensive LR formation induced by BNYVV must be the result of a disruption of the regular LR patterning, leading to the emergence of many meristematic cells that start initiation of LR development. This is evidenced by the observation that cortical cells are converted into cells with meristematic activity in BNYVV-infected LRs [83]. Within the transcriptional network, we identified several upregulated TFs limited to BNYVV (but not found in the BSBMV dataset) and associated with cell specification and proliferation. Among all differentially expressed TFs, AP2 ERF BBM, associated with cell proliferation and morphogenesis during embryogenesis [54], displayed the highest number of potential target genes as indicated by the TF enrichment analysis. Our data support the idea that the extensive LR formation is due to the conversion of root cells into meristematic cells that develop LR. Moreover, BSBMV seems to have lost the ability to induce these genes responsible for the transformation.

The LR organogenesis starts from pericycle cells which are transformed into founder cells and continue to develop into lateral roots by anticlinal and asymmetric division [24]. This transition requires a local accumulation of auxin in pericycle cells [84]. Several tryptophan-dependent pathways exist for auxin production [51], but their role during virus infection is unknown. Here, we showed that the IPyA and TRA auxin biosynthesis pathways are activated in virus-infected plants. Both pathways were more strongly activated by BNYVV, and the subsequent higher need for the precursor l-tryptophan was followed by a higher expression of a tryptophan synthetase responsible for the biosynthesis of l-tryptophan. Moreover, inactivation of auxin through conjugation with amino acid residues of proteins was strongly inhibited in BNYVV-infected plants as indicated by a downregulation of *GH3.1*. It is very likely that the stronger activation of auxin biosynthesis as well as the repression of inactivation pathways in BNYVV-infected plants probably leads to a higher level of active auxin compared to BSBMV. This is supported by the higher number of auxin-responsive genes with significantly altered expression upon BNYVV infection. Therefore, we suggest that the early disruption of the auxin metabolism represents the first step in establishing the rhizomania disease-specific root phenotype.

An important component of the auxin-triggered LR development is the LBD transcriptional network [57,58,85,86]. In a previous study, we showed that *LBD16*, *LBD18* and *LBD29* are auxin-inducible genes that are strongly upregulated upon soil-mediated infection with BNYVV [9]. Here, we identified seven differentially expressed LBDs, five of which were exclusively upregulated in BNYVV-infected plants. The LBD transcriptional network displays a dynamic expression during rhizomania disease development with higher mRNA expression levels at earlier stages of virus infection [9]. Thus, the late sampling point (6 weeks post inoculation) might explain the low expression level of *LBD16* and *LBD29* in this study. Moreover, virus-induced changes that occur early after infection are not detected here. The LR formation is initiated by an auxin signal that triggers founder cell specification and cell cycle activation of xylem pole-positioned pericycle cells. Among the upregulated LBDs in this study, LBD18 and LBD33 play a prominent role in this process, as they act in conjunction as transcriptional activator of *E2Fa* [58]. The TF E2Fa regulates the asymmetric cell division during LR initiation. It is suggested that the induction of *E2Fa* through LBDs represents a principle mechanism for auxin-dependent cell cycle activation. The strong activation of the LBD transcriptional network in conjunction with *E2Fa* by BNYVV, as observed in this study, was not found in BSBMV-infected plants, highlighting its importance for rhizomania disease development.

Among the identified DEGs in BNYVV-infected plants, the number of genes associated with cell wall organization or biogenesis was more than two-fold higher as compared to BSBMV. The EXPs are an important downstream component of the LBD network, as they mediate cell wall loosening during plant developmental processes. LBD18 acts as a transcriptional activator of *EXP14* and *EXP17*, both of which are crucial for LR formation [59,60]. Whereas the genome of sugar beet lacks *Arabidopsis EXP14* and *EXP17* orthologues, our data suggested that their role in LR development is taken over by *EXLA1a* and *EXLA1b* [9]. Interestingly, both *EXLA1a* and *EXLA1b* (below the threshold) were upregulated by BSBMV and even more strongly by BNYVV. In our previous study, we could identify 13 differentially expressed *EXPs*, but an effect of *P*. *betae* on their expression could not be excluded as a natural BNYVV population was used. However, four *EXPs* (i.e., *EXP4L*, *EXPA7*, *EXLA1a* and *EXLA1b*) were suggested to be important for rhizomania disease development [9]. Interestingly, the same set of *EXPs* was also strongly induced in the present study. Moreover, we identified seven additional *EXPs* (i.e., *EXPB15L*, *EXPA10*, *EXPB3*, *EXPA10L*-2, *EXPA4a*, *EXLA3c*, *EXLA3b*) that were induced upon infection with BNYVV and, in part, also by BSBMV. Several studies underlined the importance of EXPs for LR formation, but their cell-specific role during LR development is mainly unknown. Recently, it has been shown that EXPA1 controls anticlinal asymmetric pericycle cell divisions and is involved in pericycle cell wall re-modelling during LR initiation in *A*. *thaliana* [87]. Interestingly, *EXPA1* from *A*. *thaliana* has the highest similarity to *EXPA10* from sugar beet [9]. On the phylogenetic tree, *EXPA1* and *EXPA10* form a separate branch within clade I [9,25,88]. In rice, *EXPA10* is preferentially expressed in the root tips and required for the root cell elongation [89]. EXPs are also important host factors promoting virus replication and intercellular movement as shown for EXPA1 during *Turnip mosaic virus* infection in *N*. *benthamiana* [90]. Over-expression of *EXPA10* in rice resulted in a higher susceptibility to rice blast caused by *Magnaporthe grisea* [91]. Thus, it is very likely that the overlapping expression pattern of some *EXPs* in BNYVV- and BSBMV-infected plants reflects a similar infection strategy shared by both viruses.

The transcriptome analysis revealed a strong defence response of sugar beet towards BNYVV and BSBMV infection since DEGs belonging to the GO term “response to stress” were significantly enriched in both datasets. One important defence mechanism is inducible barriers made of lignin, preventing systemic spread of pathogens within the vascular tissues [92,93]. Lignin production in plants is nearly exclusively based on the phenylpropanoid pathway [94]. This pathway was significantly enriched in plants infected with BNYVV and BSBMV including transcripts of enzymes catalysing the production of various lignin compounds. Furthermore, the transcriptional network associated with defence response was also altered by both viruses, particularly TFs belonging to the families *AP2*, *bHLH*, *bZIP*, *ERF*, *MYB*, *NAC* and *WRKY* that often regulate defence genes. This is also supported by the high expression of several genes encoding PR proteins in BNYVV- and BSBMV-infected plants. Interestingly, the log2 fold change of these genes was always higher in BNYVV-infected plants which indicates a stronger defence response. Upregulation of PR genes has also been reported in *N*. *benthamiana* plants infected with BNYVV [95]. Activation of PR genes might suggest a SA- or JA-mediated defence, as they are regulated by both SA and JA. However, the observed downregulation of *SABP2*, which counteracts the SA-mediated defence response, suggests that BNYVV and BSBMV interfere with SA signalling. In tobacco, silencing of *SABP2* decreased local resistance to *Tobacco mosaic virus*, expression of *PR-1* and development of systemic acquired resistance [96]. Furthermore, several components of the ET pathway were strongly suppressed, particularly *ERF1*. It has been suggested that ERF1 is a key element in the integration ET and JA pathways for the regulation of defence response genes [77]. In wheat, overexpression of *ERF1* increased the resistance to *Rhizoctonia cerealis*, whereas downregulation had an opposite effect [97]. Thus, the strong downregulation of *ERF1* by BNYVV and BSBMV probably disturbs the defence response and increases the susceptibility of sugar beet to the viruses. Moreover, *ERF1* was more strongly suppressed by BSBMV which might result in a greater susceptibility. Interestingly, the number of reads obtained for BSBMV ranged between 23 and 25% and for BNYVV only between 3 and 7%. A sequencing artefact is very unlikely, since both viruses share a similar genome size. Thus, it might indicate a higher virus replication of BSBMV in LRs. This result should be confirmed by quantification of BNYVV and BSBMV with an ELISA assay using an antibody targeting both viruses.

To conclude, our study provides the first large overview of genes specifically altered in sugar beet during infection with BNYVV and BSBMV. The comparative transcriptomic analysis highlighted contrasting expression profiles of DEGs in BNYVV and BSBMV infected roots—the organ where rhizomania disease is manifested. Surprisingly, auxin biosynthesis and downstream signalling networks leading to auxin triggered LR development were, at least in some part, also altered by BSBMV, but these changes appeared to be insufficient to induce LR formation. Subsequent studies are now required in which genes specifically regulated by BNYVV are functionally analysed for their particular role during the manifestation of rhizomania disease symptoms. Such experiments must be conducted in sugar beet, since excessive LR formation is not induced in the experimental hosts *B*. *macrocarpa* and *N*. *bethamiana*. Virus-induced gene silencing (VIGS) would be a promising approach, but a viral vector for silencing root-specific genes in sugar beet has not been developed so far. Furthermore, a direct interaction with an AUX/IAA protein involved in auxin-regulated gene expression has been demonstrated only for BNYVV [9]. It remains to be shown whether BSBMV is also able to interact with AUX/IAA proteins.

## Figures and Tables

**Table 1:** Summary of raw Illumina sequencing and filtered reads after trimming and alignment of reads to virus and sugar beet genome.

Virus	Repetition	Sequenced Reads	Filtered Reads (%)	Mapping Virus (%)	Mapping Sugar Beet (%)
BNYVV	1	21,958,505	99.99	4.12	79.97
	2	21,892,827	99.98	7.32	77.43
	3	23,448,634	99.99	3.98	80.30
BSBMV	1	27,134,031	99.99	24.92	62.65
	2	24,148,407	99.99	23.22	63.35
	3	19,896,214	99.99	25.2	62.51
Healthy	1	23,734,086	100	0	84.32
	2	21,551,604	99.99	0	84.44
	3	18,678,083	99.99	0	84.13

**Table 2:** Top 20 of significantly enriched gene ontology (GO) categories of differentially expressed genes (DEGs) from BNYVV- and BSBMV-infected plants.

GOID	Term	BNYVV DEGs			BSBMV DEGs		
		Total	Up	Down	Total	Up	Down
GO:0048046	apoplast	99	75	24	54	36	18
GO:0003824	catalytic activity	883	657	226	480	311	169
GO:0071944	cell periphery	531	393	138	282	181	101
GO:0005618	cell wall	173	123	50	102	64	38
GO:0071554	cell wall organization or biogenesis	128	112	16	57	50	7
GO:0030312	external encapsulating structure	173	123	50	102	64	38
GO:0005576	extracellular region	387	301	86	216	155	61
GO:0009813	flavonoid biosynthetic process	45	32	13	29	14	15
GO:0009812	flavonoid metabolic process	52	36	16	33	16	17
GO:0016020	membrane	774	566	208	430	266	164
GO:0008152	metabolic process	991	711	280	542	330	212
GO:0055114	oxidation-reduction process	221	148	73	127	72	55
GO:0016491	oxidoreductase activity	217	151	66	128	78	50
GO:0009505	plant-type cell wall	99	72	27	59	36	23
GO:0009628	response to abiotic stimulus	246	133	113	159	66	93
GO:0042221	response to chemical	305	189	116	179	77	102
GO:1901700	response to oxygen-containing compound	199	125	74	113	49	64
GO:0050896	response to stimulus	624	398	226	371	174	197
GO:0006950	response to stress	405	263	142	249	118	131
GO:0044763	single-organism cellular process	663	469	194	373	226	147
GO:0044710	single-organism metabolic process	489	344	145	272	164	108
GO:0044699	single-organism process	961	690	271	529	325	204

**Table 3:** Significantly enriched KEGG pathways identified in BNYVV- and BSBMV-infected plants. *p*-Values lower than 0.05 are highlighted in bold letters.

KEGG Pathway	BNYVV		BSBMV	
	Genes	*p*-Value	Genes	*p*-Value
Phenylpropanoid biosynthesis	48	**2.55 × 10^−11^**	24	**3.90 × 10^−05^**
Biosynthesis of secondary metabolites	157	**9.85 × 10^−11^**	82	**4.00 × 10^−05^**
Metabolic pathways	236	**9.39 × 10^−10^**	127	**4.00 × 10^−05^**
Flavonoid biosynthesis	10	**0.0011**	5	0.0572
Starch and sucrose metabolism	32	**0.0080**	16	0.1663
Glycolysis/gluconeogenesis	21	**0.0165**	10	0.2386
Steroid biosynthesis	10	**0.0178**	3	0.4754
Pentose and glucuronate interconversions	16	**0.0205**	6	0.3923
Stilbenoid, diarylheptanoid and gingerol biosynthesis	11	**0.0271**	3	0.5831
Tyrosine metabolism	10	**0.0290**	4	0.3607
Alanine, aspartate and glutamate metabolism	11	**0.0295**	3	0.5831
Circadian rhythm-plant	9	**0.0388**	7	**0.0314**
Sesquiterpenoid and triterpenoid biosynthesis	7	**0.0391**	5	**0.0633**
Arginine and proline metabolism	11	**0.0443**	6	0.2386

**Table 4:** Log2 fold change (log2FC) of differentially expressed *lateral organ boundaries domain* (*LBDs*) and *expansin* (*EXPs*) genes in BNYVV- and BSBMV-infected plants. The data that are statistically supported (*p* > 0.05) with a log2FC > 2 are highlighted in bold.

NCBI GENE ID	Gene Name	BNYVV log2FC	*p*-Value	BSBMV log2FC	*p*-Value
***LBDs***					
104904514	*LBD 33*	**4.82**	**0.0000**	0.57	0.6849
104890156	*LBD 6*	**4.05**	**0.0000**	1.09	0.4096
104905420	*LBD 19*	**2.44**	**0.0000**	1.14	0.1231
104908367	*LBD 15*	**2.43**	**0.0000**	0.88	0.1850
104905421	*LBD 18*	**2.30**	**0.0000**	0.83	0.1930
104903105	*LBD 21*	**−0.70**	0.5803	**−2.32**	**0.0316**
104906092	*LBD 41*	**−2.77**	**0.0000**	**−2.42**	**0.0000**
104893389	*LBD 20*	**−3.42**	**0.0000**	**−2.72**	**0.0015**
***EXPs***					
104905343	*EXPB15L*	**6.31**	**0.0000**	**3.48**	**0.0001**
104892376	*EXPA7*	**4.64**	**0.0000**	2.03	0.0646
104893284	*EXPA7L*	**4.39**	**0.0001**	2.11	0.0771
104904256	*EXPA4c*	**3.52**	**0.0000**	**2.85**	**0.0000**
104887636	*EXPA10*	**3.07**	**0.0005**	**3.65**	**0.0000**
104903052	*EXPB3*	**3.05**	**0.0000**	**3.06**	**0.0000**
104903845	*EXLA1b*	**2.9**	**0.0000**	1.71	0.0456
104894031	*EXPA10L-2*	**2.43**	**0.0002**	**2.34**	**0.0005**
104887634	*EXPA4a*	**2.14**	**0.0021**	**2.02**	**0.0063**
104903843	*EXLA1a*	**2.09**	**0.0005**	**1.76**	**0.0108**
104887635	*EXPA2L*	1.43	0.2301	**2.95**	**0.0008**
104892824	*EXLB1a*	**−2.35**	**0.0000**	**−2.09**	**0.0000**
104908292	*EXLB1c*	**−3.30**	**0.0001**	−1.92	0.0523
104904178	*EXLA3c*	**−5.5**	**0.0000**	−3.63	1.0000
104904176	*EXLA3b*	**−6.57**	**0.0000**	−2.22	0.0606

**Table 5:** Overview of differentially expressed transcription factors identified in BNYVV- and BSBMV-infected plants.

Transcription Factor Family	Downregulated		Upregulated	
	BNYVV	BSBMV	BNYVV	BSBMV
AP2	1	1	3	3
ARF	0	0	1	0
ARF-B	0	0	1	0
B3	1	0	3	3
bHLH	9	6	16	5
bZIP	3	3	1	0
C2H2	2	0	5	1
C3H	2	1	1	0
CO-like	2	2	0	0
DBB	1	1	0	0
DoF	1	1	4	0
E2F/DP	0	0	1	0
ERF	5	6	11	4
FAR1	1	1	0	0
G2-like	2	0	0	0
GATA	0	0	1	1
GRAS	0	0	2	1
HD-ZIP	3	0	2	1
HRT-like	0	0	1	1
HSF	1	1	0	0
LBD	2	3	5	0
MIKC_MADS	3	1	3	0
MYB	1	2	8	3
MYB_related	1	0	3	2
NAC	0	1	8	0
NF-YA	1	0	0	0
NF-YC	0	0	0	1
RAV	1	2	0	0
TALE	1	0	1	0
TCP	0	0	2	0
Trihelix	1	1	1	0
WOX	0	0	2	0
WRKY	2	4	5	1
ZF-HD	0	0	1	0

## Supplementary Materials

The following are available online at https://www.mdpi.com/1999-4915/12/1/76/s1, Figure S1: Phenylpropanoid biosynthesis pathway of sugar beet retrieved from the KEGG database for BNYVV. Upregulated components of the pathway are highlighted in red. Figure S2: Phenylpropanoid biosynthesis pathway of sugar beet retrieved from the KEGG database for BSBMV. Upregulated components of the pathway are highlighted in red. Table S1: List of primers that were used for gene expression analysis by RT-qPCR. Table S2: Validation of RNA-Seq by RT-qPCR. Log2 fold change values were calculated relative to the healthy control. Table S3: Log2 fold change of genes (*p* < 0.05) identified in BNYVV infected plants. Table S4: Log2 fold change of genes (*p* < 0.05) identified in BSBMV infected plants. Table S5: Log2 fold change of differentially expressed genes (log2 fold change > 2 or < −2; *p*-value < 0.05) identified in BNYVV infected plants., Table S6: Log2 fold change of differentially expressed genes (log2 fold change > 2 or < −2; *p*-value < 0.05) identified in BSBMV infected plants. Table S7: Log2 fold change of auxin-responsive genes differentially expressed in BNYVV infected plants., Table S8: Transcription factor (TF) enrichment analysis using differentially expressed TF (log2 fold change > 2 or < −2) identified in BNYVV and BSBMV infected plants. Table S9: Log2 fold change of plant defence-related transcription factors (TFs) differentially expressed in BNYVV and BSBMV infected plants.
