# Supplementary figures and images for: Comparative Transcriptome Analysis Provides Molecular Insights into the Interaction of Beet necrotic yellow vein virus and Beet soil-borne mosaic virus with Their Host Sugar Beet
Source: Viruses. 2020 Jan 8;12(1):76. doi: 10.3390/v12010076 (PMC7019549; doi:10.3390/v12010076)

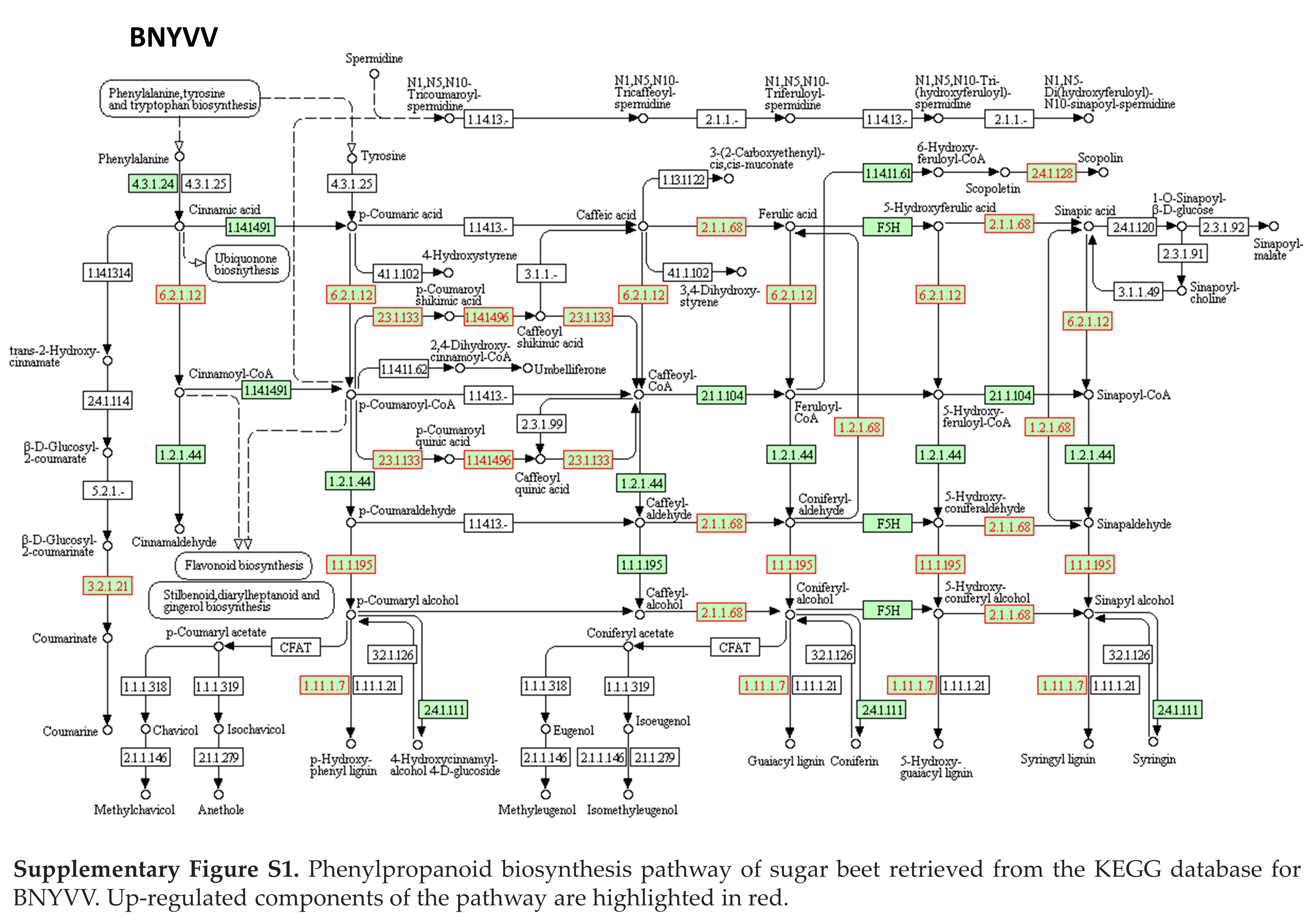

Supplement: Supplementary file 1 [file viruses-12-00076-s001.zip › Supplementary Figure S1.tif]

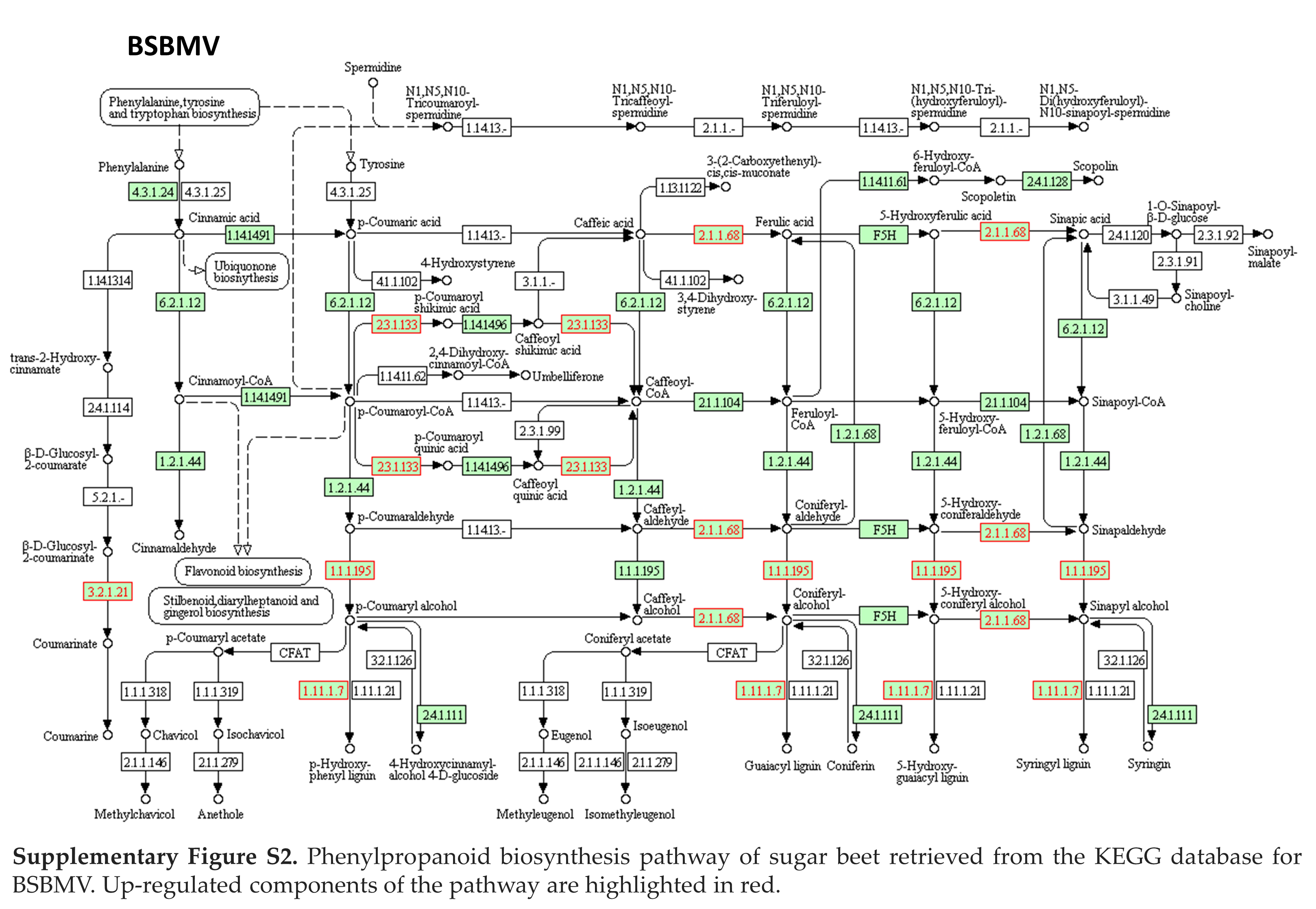

Supplement: Supplementary file 1 [file viruses-12-00076-s001.zip › Supplementary Figure S2.tif]
